# Past hybridization between two East Asian long-tailed tits (*Aegithalos bonvaloti* and *A. fuliginosus*)

**DOI:** 10.1186/1742-9994-11-40

**Published:** 2014-05-16

**Authors:** Wenjuan Wang, Chuanyin Dai, Per Alström, Chunlan Zhang, Yanhua Qu, Shou-Hsien Li, Xiaojun Yang, Na Zhao, Gang Song, Fumin Lei

**Affiliations:** 1 Key Laboratory of Zoological Systematics and Evolution, Institute of Zoology, Chinese Academy of Sciences, No.1 Beichen West Road, Chaoyang District, 100101 Beijing, China; 2 Center for Watershed Ecology, Institute of Life Science, Nanchang University, 330031 Nanchang, China; 3 School of Chemistry and Life Sciences, Guizhou Normal College, 550001 Guiyang, China; 4 Swedish Species Information Centre, Swedish University of Agricultural Sciences, SE-75007 Uppsala, Sweden; 5 Department of Life Science, National Taiwan Normal University, Taipei, Taiwan; 6 State Key Laboratory of Genetic Resources and Evolution, Kunming Institute of Zoology, Chinese Academy of Sciences, 650223 Kunming, China; 7 Key Laboratory of Ecological Impacts of Hydraulic-Projects and Restoration of Aquatic Ecosystem of Ministry of Water Resources, Institute of Hydroecology, Ministry of Water Resources and Chinese Academy of Sciences, 430079 Wuhan, China

**Keywords:** Incomplete lineage sorting, Introgression, Isolation-with-migration, Multilocus analyses, Ecological niche modelling

## Abstract

**Introduction:**

Incomplete lineage sorting and hybridization are two major nonexclusive causes of haplotype sharing between species. Distinguishing between these two processes is notoriously difficult as they can generate similar genetic signatures. Previous studies revealed that the mitochondrial DNA (mtDNA) differentiation between two East Asian long-tailed tits (*Aegithalos bonvaloti* and *A. fuliginosus*) was extremely low, even lower than intraspecific differentiation in some other long-tailed tits. Using a combination of multilocus and coalescent analyses, we explored the causes of the anomalous lack of mtDNA differentiation between the two species.

**Results:**

The mtDNA divergence between the two species was shallow, while the nuclear DNA (nuDNA) divergence was considerably deeper. The IMa analyses based on the mtDNA dataset suggested relatively high gene flow from *A. fuliginosus* to *A. bonvaloti*, while negligible gene flow in the opposite direction. In contrast to mtDNA, the migration rates at autosomal and Z-linked nuDNA loci were negligible or much lower. The NEWHYBRIDS analysis assigned all individuals except one to pure parental species with high posterior probability. The Bayesian skyline plot showed that both species underwent population expansions during the Last Glacial Maximum (LGM), and the ecological niche modelling suggested that their ranges overlapped more during the LGM than at present.

**Conclusions:**

We suggest that historical hybridization, in combination with selective sweep and/or genetic drift might be the main causes of the extremely low mtDNA differentiation between the two species. The hybridization probably occurred mainly between *A. fuliginosus* females and *A. bonvaloti* males. The LGM distribution expansion might have facilitated hybridization, while the post-LGM distribution contraction could have facilitated some mtDNA sorting. Ongoing hybridization between the two species might be very limited, but further studies with more samples from the contact zone are needed to test this conclusion.

## Introduction

It is widely recognized that gene trees can differ from the species phylogeny, and gene genealogies usually proceed through various stages of non-monophyly during the early stages of their divergence history [1-6]. In a survey of published mitochondrial analyses, Funk & Omland [4] identified species-level non-monophyly in 23% of the more than 2300 assayed species. Hybridization and incomplete lineage sorting (ILS) are considered to be two of the major, nonexclusive causes of species-level non-monophyly [4,7,8]. Hybridization between species may result in the incorporation of alleles from one species into the gene pool of another [9]. When the first generation hybrids backcross with at least one of the parental species, introgression occurs. Alternatively, ILS may also result in species-level non-monophyly if species divergence was too recent for ancestral polymorphisms to have sorted to reciprocal monophyly. Distinguishing between hybridization and ILS is notoriously difficult, because they leave similar genetic signatures [10-12].

A commonly used approach to distinguish between hybridization and ILS is comparing genetic diversity between mitochondrial DNA (mtDNA) and nuclear DNA (nuDNA). The presence of fixed differences between species in nuDNA despite mtDNA haplotype sharing, or deeper divergences in nuDNA than in mtDNA, are often interpreted as evidence of mtDNA migration [13-17]. However, the stochasticity of the coalescent process (i.e. randomness of mutation and genetic drift) and the potential for locus-specific effects such as selection or recombination, both of which might result in fixed base pair differences between species at some loci while shared haplotypes at other loci, prevent strong conclusions [18]. Massive hybridization over a long period could also homogenize nuDNA, making conclusions more difficult to draw [19]. Most importantly, this approach cannot exclude the possibility of a combined effect of both hybridization and ILS. Therefore, quantitative evaluation of the likelihood of alternative hypotheses is needed to draw robust conclusions [15,20].

The development of the isolation-with-migration (IM) coalescent model, which takes into account inheritance models of markers and the stochasticity of the coalescent process, allows us to rigorously test explicit hypotheses [21-23]. The IM model is usually used to test two hypotheses related to divergence processes. The first hypothesis is a strict isolation model whereby speciation resulted from allopatric divergence without subsequent gene flow. The second hypothesis is an isolation with migration model that allows speciation despite the presence of gene flow. Non-monophyly can be attributed to ILS when the IM model supports the strict isolation model. In contrast, if the IM model supports the isolation with migration model, introgression may be necessary to explain non-monophyly. Additionally, the IM model could also be used to reveal introgression rates and gene flow directions at different marker systems [24-26], and identify candidate gene for reproductive isolation [27,28].

**Figure 1:**
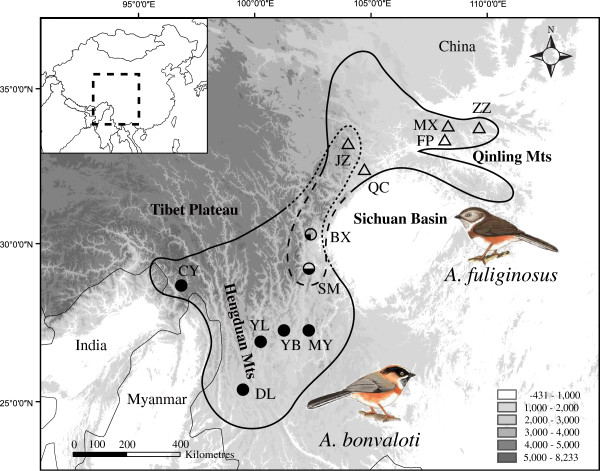
**Map of the study area and sampling locations.** Solid line represents the distribution of *Aegithalos bonvaloti* and *A. fuliginosus*. Dotted line represents the approximate contact zone. Circles and triangles show the sampling locations of *A. bonvaloti* and *A. fuliginosus*, respectively. The colours of circles and triangles correspond to mitochondrial DNA (mtDNA) origin, with white for *A. fuliginosus* haplotypes and black for *A. bonvaloti* haplotypes. Elevation is represented by shading: lower elevations are shown lighter and higher elevations are shown darker. Insert: The dotted box represents the study region.

*Aegithalos bonvaloti* (Black-browed Tit) and *A. fuliginosus* (Sooty Tit) are two East Asian endemic long-tailed tits. The two species are closely related to each other [29,30]. *A. bonvaloti* is distributed from southwest China to extreme northeast Myanmar, and *A. fuliginosus* is restricted to the mountainous areas of central China (Figure 1; [31,32]). The two species are parapatrically distributed, with distributions partially overlapping to the west of the Sichuan Basin. *A. bonvaloti* is distributed from about 1500 – 4400 m, and *A. fuliginosus* from about 1000 – 2600 m [33]. Two subspecies are recognized in *A. bonvaloti*: *bonvaloti* in the southern part of the species’ range and a doubtful subspecies *obscuratus* (see Discussion for details) in the northern part [30]. In contrast, *A. fuliginosus* is monotypic. The plumage differences between the two species are pronounced (cf. Figure 1). Intriguingly, the mtDNA divergence between them is extremely low (cytochrome-*b* uncorrected *p*-distance: 0.002), even lower than intraspecific differentiation within some other long-tailed tit species [29,30]. Päckert et al. [30] suggested that *A. bonvaloti* and *A. fuliginosus* may “represent just distinct morphotypes at an intraspecific differentiation level”. However, both Dai et al. [29] and Päckert et al. [30] included only one sample of *A. bonvaloti* and fewer than six samples of *A. fuliginosus*, so whether their results represent the true level of differentiation is unknown.

In the present study, we explored mtDNA diversification between *A. bonvaloti* and *A. fuliginosus* using multiple samples throughout the range of both species. We first investigated whether the previously reported extremely low mtDNA divergence between them is also evident under larger sample sizes. As this was confirmed, we compared the genetic variation between mtDNA and nuDNA datasets, and utilized the IM model to test whether this was caused by ILS or hybridization. Finding support for historical hybridization, we tried to explore the interspecific gene flow pattern and related explanations. Finally, as it is uncertain whether the individuals representing intermediate phenotypes from the contact zone are of hybrid origin or represent a distinct subspecies *A. b. obsuratus*, we further investigate if contemporary hybridization occurs between the two species.

## Results

### Genetic polymorphism and species phylogeny

A total of 26 and 31 individuals were sampled for *A. bonvaloti* and *A. fuliginosus*, respectively (Figure 1 and Table 1). Two mtDNA fragments, three autosomal nuDNA loci and three Z-linked nuDNA loci were utilized. The mtDNA fragments were *cytb* (cytochrome *b*) and *ND2* (NADH dehydrogenase subunit 2). The three autosomal nuDNA loci were *MB* (myoglobin, intron 2), *TGFB2* (transforming growth factor beta-2, intron 5) and *RAG1* (recombination activating protein 1). The three Z-linked nuDNA loci were *BRM15* (brahma protein, intron 15), *CHDa* (chromo-helicase-DNA-binding protein, intron a) and *VLDLR9* (very low density lipoprotein receptor, intron 9).

**Table 1:** Mitochondrial DNA nucleotide polymorphism at each sampling site

**Population code**	**Location**	**Longitude**	**Latitude**	***n***	***nh***	π	***h***
*A. bonvaloti*				26	8	0.00107	0.81
CY	Chayu	97.084	28.563	12	3	0.00041	0.67
YL	Yulong	100.261	27.097	1	1	—	—
DL	Dali	100.000	25.417	2	2	0.00105	1.00
YB	Yanbian	101.550	27.100	3	3	0.00105	1.00
MY	Miyi	101.899	27.127	1	1	—	—
SM	Shimian	102.338	29.100	2	2	0.00157	1.00
BX	Baoxing	102.811	30.370	5	2	0.00063	0.40
*A. fuliginosus*				31	7	0.00051	0.61
JZ	Jiuzhaigou	103.807	33.309	12	4	0.00052	0.68
QC	Qingchuan	104.736	32.592	5	3	0.00052	0.80
FP	Foping	107.768	33.596	8	1	0.00000	0.00
MX	Moxi	107.796	34.019	2	2	0.00210	1.00
ZZ	Zhouzhi	104.171	33.697	4	3	0.00052	0.83

*n*, sample size; *nh*, number of haplotypes; π, nucleotide diversity; *h*, haplotype diversity.

The absence of ambiguous sites and stop codons suggested that these mtDNA sequences were not “numts” [34]. The combined mtDNA alignment contained 21 polymorphic sites, which defined 14 haplotypes. The mtDNA haplotype diversity of each population ranged from 0.40 to 1.00 in *A. bonvaloti* and from 0.68 to 1.00 in *A. fuliginosus*, and the nucleotide diversity of each population ranged from 0.00041 to 0.00157 in *A. bonvaloti* and from 0 to 0.0021 in *A. fuliginosus* (Table 1). For the nuDNA loci, the number of haplotypes, haplotype diversities and nucleotide diversities were similar between the two species (Table 2). The results of multilocus HKA test demonstrated no significant departure from neutrality for all loci [sum of deviations = 18.40, degrees of freedom (df) = 12, *P* = 0.10]. Tajima’s *D* test [35] revealed that most loci did not depart significantly from neutrality, except for *ND2* and the entire mtDNA alignment in *A. fuliginosus*. The assumption of neutrality for all loci could not be rejected by Fu and Li’s *D* test [36].

**Table 2:** Summary statistics for each gene region and values of neutrality tests

		***cytb***	***ND2***	**mtDNA**	***MB***	***TGFB2***	***RAG1***	***BRM15***	***CHDa***	***VLDLR9***
										
full length		929	979	1908	737	600	867	236	574	274
*Rm*		—	—	—	0	0	3	1	1	0
non-recombining length		—	—	—	—	—	435	97	286	—
N	*A. bonvaloti*	26	26	26	40	52	50	50	44	44
	*A. fuliginosus*	31	31	31	18	56	42	48	62	56
*s*	*A. bonvaloti*	7	4	11	1	2	5	8	2	5
	*A. fuliginosus*	2	8	10	0	3	9	7	0	1
*nh*	*A. bonvaloti*	8	5	8	2	3	7	11	4	6
	*A. fuliginosus*	3	6	7	1	4	13	10	1	2
π	*A. bonvaloti*	0.0015	0.0006	0.0011	0.0001	0.0004	0.0018	0.0056	0.0014	0.0014
	*A. fuliginosus*	0.0002	0.0008	0.0005	—	0.0006	0.0016	0.0050	—	0.0001
*h*	*A. bonvaloti*	0.81	0.55	0.81	0.10	0.21	0.80	0.77	0.61	0.36
	*A. fuliginosus*	0.19	0.53	0.61	—	0.36	0.76	0.71	—	0.04
Tajima’s *D*	*A. bonvaloti*	-0.70	-1.08	**-**0.98	-0.84	-0.91	1.01	-0.70	1.47	-1.64
	*A. fuliginosus*	-1.26	-1.82*	-1.91*	—	-0.84	-0.92	-0.69	—	-1.09
Fu and Li’s *D*	*A. bonvaloti*	-0.06	-0.90	-0.48	0.56	-0.9	1.10	-0.13	0.76	-1.72
	*A. fuliginosus*	-0.75	-2.51	-2.40	—	-0.44	-1.18	0.47	—	-1.87
*p*-distance	*A. bonvaloti–A. fuliginosus*	0.002	0.001	0.002	0.003	0.004	0.002	0.005	0.008	0.001

*Rm*, minimum number of recombination events; N, sample size of each species; *s*, polymorphic sites; *nh*, number of haplotypes; π, nucleotide diversity; *h*, haplotype diversity; *p*-distance, uncorrected *p*-distance between *Aegithalos bonvaloti* and *A. fuliginosus;* *, *P*< 0.05*. CHDa*, *VLDLR9* and *BRM15* are Z-linked loci, and *TGFB2*, *RAG1* and *MB* are autosomal loci.

**Figure 2:**
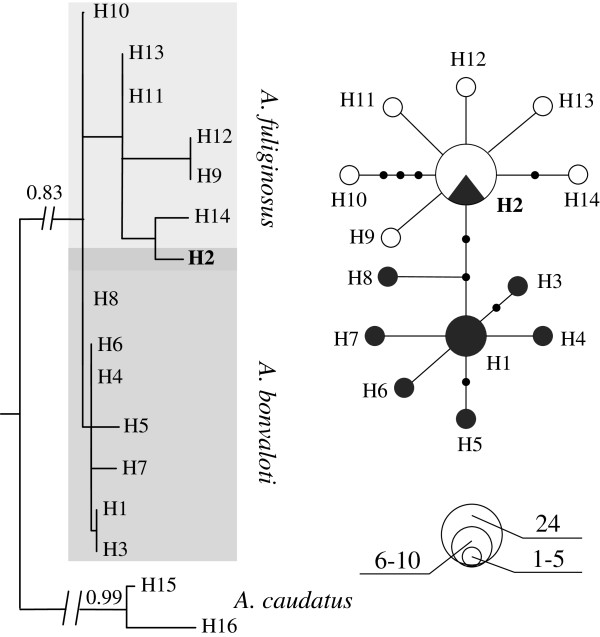
**Maximum clade credibility Bayesian tree and haplotype network of mitochondrial DNA.** Branch support values higher than 0.80 are shown above nodes. Each circle represents a haplotype, and the size of the circle is proportional to that haplotype’s frequency. Dots represent unsampled haplotypes. Haplotypes are coloured according to taxon: black for *Aegithalos bonvaloti* and white for *A. fuliginosus. H2* is the most widely distributed haplotype shared by 19 individuals of *A. fuliginosus* and 5 individuals of *A. bonvaloti.*

**Figure 3:**
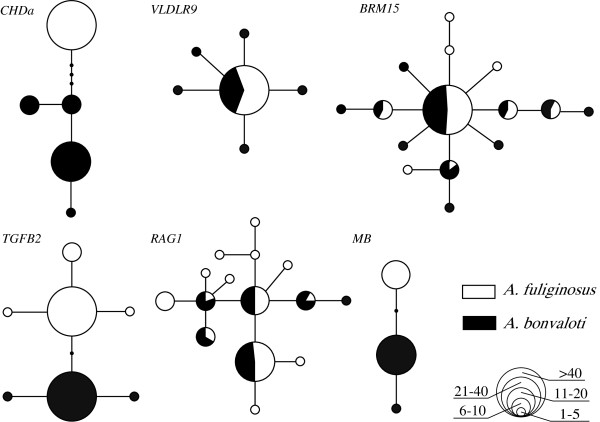
**Haplotype networks constructed with nuclear loci.** The three loci in the upper row (*CHDa*, *VLDLR9* and *BRM15*) are Z-linked, whereas the three in the lower row (*TGFB2*, *RAG1* and *MB*) are autosomal. Each circle represents a haplotype, and the size of the circle is proportional to that haplotype’s frequency. Dots represent unsampled haplotypes. Haplotypes are coloured according to taxon: black for *Aegithalos bonvaloti* and white for *A. fuliginosus*.

The two species were not reciprocally monophyletic in the mtDNA gene tree (Figure 2). The most abundant mtDNA haplotype was shared by 19 individuals of *A. fuliginosus* and 5 individuals of *A. bonvaloti*. No other mitochondrial haplotype was shared between the two species. Nuclear haplotypes were shared between the two species at *BRM15*, *VLDLR9* and *RAG1* (Figure 3). However, fixed base pair differences were found at *MB*, *TGFB2* and *CHDa*.

In order to evaluate whether the mtDNA divergence is reduced relative to nuDNA, we calculated the uncorrected *p*-distance between the two species at each locus, and the mtDNA to nuDNA divergence ratios between the two species and other *Aegithalos* species pairs. The mean uncorrected *p*-distance between *A. bonvaloti* and *A. fuliginosus* was 0.002 for mtDNA, whereas it was on average twice as high for the nuDNA loci (mean 0.004; range 0.001 to 0.008; Table 2). The mean ratio of mtDNA to nuDNA divergence of *A. bonvaloti* and *A. fuliginosus* (0.4) was considerably lower than in other *Aegithalos* species pairs (mean 7.6; range 4.4 to 10.7; Additional file 1).

### Coalescent estimates of gene flow

We applied the coalescent-based IM model implemented in the program IMa [22] to distinguish between ILS and hybridization. This method co-estimates several parameters, including effective population size, divergence time and migration rate. In order to reduce the risk that the IMa would confuse genetic homoplasy with hybridization, we conducted two kinds of analyses, one with all individuals, and the other with only allopatric individuals. If the analysis based on all individuals reveals interspecific gene flow, while the analysis based on only allopatric individuals reveals near-zero gene flow, this suggests that the interspecific gene flow is restricted to the hybrid zone, and that hybridization occurs between the species. However, if both analyses show interspecific gene flow, this suggests that ILS might be needed to explain the genetic pattern. To facilitate comparisons of introgression estimates between different marker systems, we conducted analyses based on three separate datasets: one with the three Z-linked loci, one with the three autosomal loci, and one with the mtDNA.

**Figure 4:**
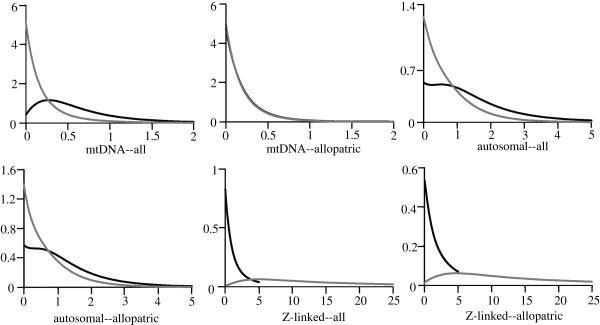
**The marginal posterior probability density distributions of migration rates (*m*).** All parameter estimates are scaled to the mutation rate. The black line represents migration rate from *Aegithalos fuliginosus* to *A. bonvaloti*. The grey line represents migration rate from *A. bonvaloti* to *A. fuliginosus*. “all” and “allopatric” mean IM analyses based on all individuals and allopatric individuals, respectively.

The IMa analyses produced posterior distributions of migration rate and effective population size parameters with clear peaks and bounds within the prior distribution (Figure 4 and Additional file 2), which suggests that these results were reliable. The posterior distributions of the divergence time parameters also had clear peaks, however the upper bounds did not return to zero within the prior distribution. The low levels of variation in the analyzed loci may prevent the divergence time parameters from being estimated accurately.

For the mtDNA dataset, none of the four models could be rejected (*P*> 0.05, Additional file 3). For the autosomal and Z-linked DNA datasets, the strict allopatric speciation models were rejected. For the mtDNA dataset including all individuals, the estimated migration rate from *A. fuliginosus* to *A. bonvaloti* was about 1.54 migrants per generation, while the migration rate from *A. bonvaloti* to *A. fuliginosus* was negligible (Table 3). For the mtDNA dataset based on allopatric individuals, the estimated gene flow in both directions was negligible. For the autosomal DNA dataset, the migration rates in both directions were near zero. For the Z-linked DNA dataset including all individuals, the migration rate from *A. bonvaloti* to *A. fuliginosus* was about 0.18 migrants per generation, while the migration rate from *A. fuliginosus* to *A. bonvaloti* was negligible. For the Z-linked DNA dataset based on allopatric individuals, the estimated migration rate from *A. bonvaloti* to *A. fuliginosus* was 0.15 migrants per generation, i.e. similar to that based on all individuals.

**Table 3:** Demographic parameters estimated with IMa

	**Θ**_***b***_	**Θ**_***f***_	**Θ**_***A***_	***m***_***fb***_	***m***_***bf***_	***2N***_***b***_ ***m***_***fb***_	***2N***_***f***_ ***m***_***bf***_
Mitochondrial DNA dataset from all individuals							
MLE	11.47	9.37	3.28	0.27	0.00	1.54	0.00
HPD90Lo	4.72	3.73	0.02	0.00	0.00	—	—
HPD90Hi	28.16	21.40	35.37	1.24	0.63	—	—
Mitochondrial DNA dataset from allopatric individuals							
MLE	8.12	9.19	3.09	0.00	0.00	0.00	0.00
HPD90Lo	2.98	3.48	0.02	0.00	0.00	—	—
HPD90Hi	20.05	22.65	31.01	0.55	0.55	—	—
Autosomal DNA dataset from all individuals							
MLE	0.17	0.42	0.44	0.00	0.00	0.00	0.00
HPD90Lo	0.05	0.17	0.00	0.00	0.00	—	—
HPD90Hi	0.49	0.92	4.43	2.97	1.83	—	—
Autosomal DNA dataset from allopatric individuals							
MLE	0.16	0.34	0.51	0.00	0.00	0.00	0.00
HPD90Lo	0.04	0.12	0.01	0.00	0.00	—	—
HPD90Hi	0.45	0.78	4.06	2.65	1.82	—	—
Z-linked DNA dataset from all individuals							
MLE	0.84	0.06	0.08	0.00	5.54	0.00	0.18
HPD90Lo	0.37	0.01	0.00	0.00	1.11	—	—
HPD90Hi	1.72	0.24	3.45	3.58	21.34	—	—
Z-linked DNA dataset from allopatric individuals							
MLE	0.69	0.06	0.02	0.00	4.79	0.00	0.15
HPD90Lo	0.24	0.02	0.00	0.00	0.49	—	—
HPD90Hi	1.91	0.30	2.80	3.80	20.84	—	—

MLE, maximum-likelihood estimates; HPD, 90% highest posterior density intervals (Lo, lowest value; Hi, highest value); Θ_*b*_, theta for *Aegithalos bonvaloti*; Θ_*f*_, theta for *A. fuliginosus*; Θ_*A*_, theta for ancestral species; *m*_*fb*_, migration rate per mutation from *A. fuliginosus* to *A. bonvaloti*; *m*_*bf*_, migration rate per mutation from *A. bonvaloti* to *A. fuliginosus*; 2*N*_*b*_*m*_*fb*_, the effective number of migrants per generation from *A. fuliginosus* to *A. bonvaloti*; 2*N*_*f*_*m*_*bf*_, the effective number of migrants per generation from *A. bonvaloti* to *A. fuliginosus*.

### Historical demographic and spatial dynamics

**Figure 5:**
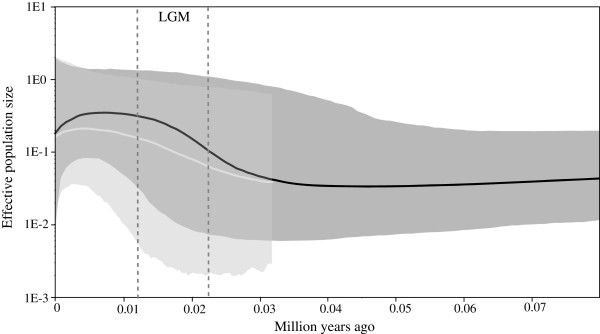
**Bayesian skyline plots representing historical demographic trends of *Aegithalos bonvaloti *and *A. fuliginosus*.** The *x*-axis is in unit of million years ago. The *y*-axis shows the scaled effective population size. The median estimate is enclosed within the 95% HPD limits. Black and grey represent *A. bonvaloti* and *A. fuliginosus*, respectively. Dotted lines show the Last Glacial Maximum (LGM).

To gain insight into the relationship between hybridization and past population dynamics, we explored the historical demographic and spatial dynamics of the two species. Mismatch distribution and Bayesian skyline plot (BSP) analyses were conducted to explore the past population dynamics. The mismatch distributions for both species were unimodal, which suggest that the two species underwent population expansions. The results of the BSP analysis suggested population expansions in both species during the Last Glacial Maximum (LGM; Figure 5).

**Figure 6:**
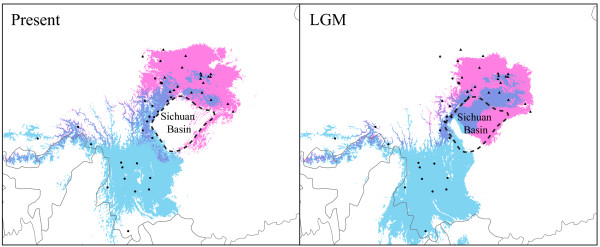
**Ecological niche models for *Aegithalos bonvaloti *and *A. fuliginosus*.** The LGM represents the Last Glacial Maximum. Circles and triangles represent the localities used to build ecological niche models for *A. bonvaloti* and *A. fuliginosus,* respectively. Blue represents the potential distribution for *A. bonvaloti*, red for *A. fuliginosus*, and dark blue for the contact zone between the two species. The dotted area shows the Sichuan Basin.

Ecological niche modelling (ENM) was used to predict the potential distributions of both species at present and at the LGM. The high AUC (area under the receiver operating characteristic curves; 0.991 for *A. fuliginosus* and 0.989 for *A. bonvaloti*) and low binomial probabilities (*P*≪ 0.0001) indicate that the ENMs for both species perform better than random predictions. The predicted present distributions are largely congruent with the species’ known distributions (Figure 6). During the LGM, the predicted distributions for both species were extended to the low elevational Sichuan Basin, resulting in potentially larger contact area between the two species. After the LGM, both species moved to high elevational areas, thus contracting the contact area.

### Contemporary gene exchange

**Figure 7:**
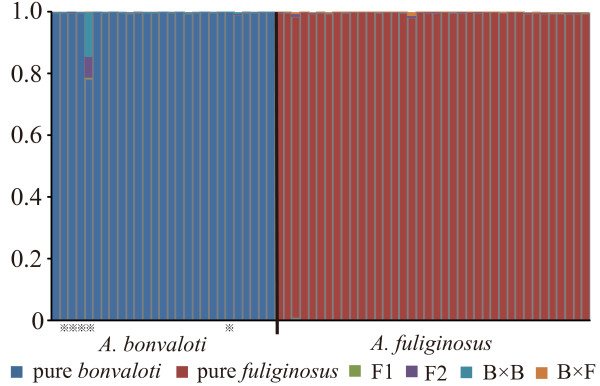
**Detection of ongoing hybridizaiton using NEWHYBRIDS.** Each histogram represents an individual. Field identification of the individuals is labeled at the bottom of each histogram. The posterior probability for each individual to be assigned as pure *Aegithalos bonvaloti*, pure *A. fuliginosus*, F1 hybrid, F2 hybrid, backcross with *A. bonvaloti* (B × B), and backcross with *A. fuliginosus* (B × F) are labeled in different colors. Asterisks indicate *A. bonvaloti* individuals with foreign mtDNA haplotype.

We used NEWHYBRIDS [37] to explore whether ongoing hybridization occurs between the two species. The NEWHYBRIDS analysis was based on the nuDNA dataset. The analysis assigned almost all individuals to pure parental species with high posterior probability (*P*> 0.99; Figure 7). Only one individual, an *A. bonvaloti* which had a foreign mtDNA haplotype, was assigned to pure *A. bonvaloti* with relatively low posterior probability (*P* = 0.78).

## Discussion

### Extremely low mtDNA divergence

Our results confirm the previous reports [29,30] of extremely low mtDNA differentiation between *A. bonvaloti* and *A. fuliginosus*, and show that this low divergence prevails across a large geographical area. The two species were not reciprocally monophyletic in the mtDNA gene tree, and there were no fixed mtDNA differences between them. Five individuals of *A. bonvaloti* from sampling sites SM and BX shared a haplotype with individuals of *A. fuliginosus* from a wide geographical range. The uncorrected *p*-distance between the two species was lower than intraspecific divergence in most other long-tailed tit species [29,30]. Moreover, the ratio of mtDNA to nuDNA divergence between the two species was much lower than in the other *Aegithalos* species pairs.

Several lines of evidence support that historical hybridization rather than ILS is the main cause of the exceptionally low mtDNA divergence between the two species. The fixed differences between the two species at *MB*, *TGFB2* and *CHDa* suggest that they have experienced a prolonged period of independent evolution. This is in agreement with the significant morphological differences between them. Although the shallow divergence and lack of reciprocal monophyly in mtDNA is compatible with recent lineage splitting, that is unlikely given the considerably deeper divergences in nuDNA. The effective population size of mtDNA is one quarter of that of nuDNA, and the mutation rate of mtDNA is generally an order of magnitude greater than that of nuDNA, thus, the mtDNA should be considerably more differentiated than the nuDNA markers [38-40]. However, this is opposite to the observed pattern in the two species. The results of the IMa analyses provide additional quantitative support for hybridization. Although the IMa analysis based on the mtDNA dataset rejected neither the strict allopatric speciation model nor the models with gene flow, migration was detected between the two species. The IMa analysis based on all individuals showed high levels of mtDNA gene flow from *A. fuliginosus* to *A. bonvaloti*, while the analysis based on allopatric individuals showed near-zero gene flow. These results suggest that the estimated gene flow is restricted to the contact zone, and that hybridization has occurred between the two species. As further support, ILS would not be expected to result in haplotype sharing exclusively in a geographical overlap zone between the two species [4,15,41].

Our IMa analyses suggested that the mtDNA gene flow was asymmetric, with high gene flow from *A. fuliginosus* to *A. bonvaloti*, and negligible gene flow in the reverse direction. In agreement with this, we found an *A. fuliginosus* mtDNA haplotype at the northernmost sampling sites within the range of *A. bonvaloti* (BX and SM), whereas only native mtDNA haplotypes among all the samples of *A. fuliginosus*. These results suggest that hybridization occurred mainly between *A. fuliginosus* females and *A. bonvaloti* males.

In general, females would prefer to mate with conspecific males rather than with heterospecific ones when given a choice, but may sometimes mate with a heterospecific male when heterospecifics dominate in numbers [42,43], possess brighter coloration [44], display more attractive courtship [45], or behave more aggressively [46,47]. A female *A. fuliginosus* in an area with predominantly *A. bonvaloti* (i.e. the southern part of the distribution of *A. fuliginosus*) might mate with a male *A. bonvaloti* as *A. bonvaloti* is more brightly coloured than *A. fuliginosus*[31,32], whereas a female *A. bonvaloti* in an area with mainly *A. fuliginosus* (i.e. the northern part of the distribution of *A. bonvaloti*) might be less likely to choose a male *A. fuliginosus* because of his duller coloration. Female-biased dispersal could also have contributed to the observed pattern, if females of *A. fuliginosus* have been more dispersive than males and more frequently dispersed southward to areas where conspecific males are uncommon. Northward dispersal of male *A. bonvaloti* could result in a similar pattern, but as the general dispersal pattern in birds is female-biased [48,49], female-biased southward dispersal of *A. fuliginosus* seems more likely.

In contrast to mtDNA, the nuDNA gene flow was limited. Strong mtDNA introgression despite limited nuDNA migration has been found in several hybrid zones [8,50-53]. This is consistent with the theoretical expectation that mtDNA is more likely than nuDNA to be transmitted across species boundaries [4,54]. As the effective population size of mtDNA is one quarter of that of nuDNA, foreign mtDNA haplotypes stand a greater chance of reaching fixation than foreign nuDNA haplotypes [4]. Moreover, mtDNA is less likely to be constrained by linkage to loci under selection than nuDNA, and is thus more susceptible to move across species boundaries [55,56].

However, we note that introgression cannot on its own explain the low mtDNA divergence between *A. bonvaloti* and *A. fuliginosus*. In view of the pronounced nuDNA divergences, the original mtDNA haplotypes should have been considerably more divergent than observed, resulting in sharing of a mixture of divergent haplotypes. As that is not the case, some process must have homogenized the mtDNA haplotypes. We suggest that a past selective sweep might have been involved. Given its role in oxidative phosphorylation, mtDNA is particularly likely to be the subject of selective sweeps, especially in organisms living in different thermal regimes [8,57-59]. The two long-tailed tits indeed occur at partly different elevations with different environments. In addition, Tajima’s *D* test revealed that the *ND2* gene and the entire mtDNA alignment in *A. fuliginosus* may be under natural selection (*P*< 0.05). In accordance with our results, natural selection on the *ND2* gene has been found in human populations [60], and has been suggested to be the source of shallow genetic structure in Willow Tit (*Parus montanus*; [61]). A very similar pattern to that found in the two long-tailed tits, which was also suggested to be the result of a selective sweep, has been observed in two widespread Palearctic buntings, Yellowhammer *Emberiza citrinella* and Pine Bunting *E. Leucocephalos*[13,17].

Genetic drift could also have increased the frequency of introgressed haplotypes, even though the introgressed haplotypes are not under positive selection [51,62,63]. Our BSP analyses revealed that the population sizes of both species were small before the LGM, which might have facilitated genetic drift.

In addition, range expansion during the LGM might have facilitated hybridization. Montane species generally undergo movements along elevational gradients to track the environmental conditions they have adapted over time [64,65], descending to lower elevational areas during glacials and becoming vicariantly isolated on different mountains during interglacials. Glacial-induced range expansion has been suggested to create great opportunities for the contact and hybridization between populations associated with different mountain systems [66,67]. The ENM analysis showed that both species underwent range expansions to the low altitude Sichuan Basin during the LGM, resulting in more extensively overlapping distribution. The larger overlapping distribution might facilitate hybridization between the two species.

Although the mtDNA divergence between *A. bonvaloti* and *A. fuliginosus* is extremely low, if the most abundant haplotype *H2* is excluded, there are fixed differences between them. This suggests that the two species have undergone more recent mtDNA sorting. Our ENM indicated that both species’ ranges contracted after the LGM, thereby reducing the horizontal contact area. The partly different elevational ranges of the two species further limited the vertical contact area. The relatively narrow overlapping distributions might have allowed only a narrow conduit for mtDNA to pass between the two species, thus facilitating sorting of mtDNA haplotypes.

### Ongoing gene exchange

Hybridization between *A. bonvaloti* and *A. fuliginosus* has been postulated based on a series of specimens representing an intermediate phenotype [30,68]. However, *A. bonvaloti* has a doubtful subspecies, *A. b. obscuratus*, which is distributed at the contact zone between *A. bonvaloti* and *A. fuliginosus. A. b. obscuratus* is slightly duller and browner than *A. b. bonvaloti*, and has been regarded as approaching *A. fuliginosus,* or to be intermediate between *A. b. bonvaloti* and *A. fuliginosus*[30,31]. It is uncertain whether the individuals representing intermediate phenotypes are hybrids or belong to *A. b. obscuratus*. Our NEWHYBRIDS analysis assigned all individuals except one to pure parental species with high posterior probability, suggesting that the ongoing gene exchange between the two species might be limited. However, we note that we included few samples from the contact zone, so more samples are needed to explore if hybridization still takes place, and to re-evaluate the status of *A. b. obscuratus*.

## Conclusions

Based on larger sample sizes than in earlier studies, we confirm that the mtDNA divergence between *A. fuliginosus* and *A. bonvaloti* is extremely low. We suggest that past hybridization in combination with a selective sweep and/or genetic drift are the main causes of this pattern. The interspecific mtDNA gene flow was species-biased, with relatively high gene flow from *A. fuliginosus* to *A. bonvaloti*, while only negligible gene flow in the reverse direction. Non-assortative mating and/or female-biased southward dispersal of *A. fuliginosus* might have contributed to the unidirectional mtDNA gene flow. In contrast to mtDNA, the interspecific nuDNA gene flow was negligible or much lower. Range expansion during the LGM might have facilitated hybridization, while post-LGM distribution contraction could have led to some sorting of mtDNA haplotypes. Our results suggest that the ongoing interspecific gene exchange might be very limited. However, more samples from the contact zone are needed to draw robust conclusions.

## Methods

### Sampling and DNA sequencing

Sample sizes for *A. bonvaloti* and *A. fuliginosus* were 26 and 31 individuals, respectively. *A. caudatus* (Long-tailed Tit), which is closely related to *A. bonvaloti* and *A. fuliginosus*[29,30], was used as outgroup. Samples were stored in 100% ethanol in the field and later at -80°C in the laboratory. Total genomic DNA was extracted from blood or muscle samples using the QIAamp DNA Mini Kit (QIAGEN) following the manufacturer’s protocol.

We amplified the two mtDNA fragments and six nuDNA loci via polymerase chain reaction (PCR). Primer pairs for *cytb* and *ND2* were H16065/L14990 and H6313/L5219, respectively [69]. PCR conditions were as follows: denaturation at 94°C for 2 min, followed by 40 cycles at 93°C for 1 min, annealing temperature (49°C for *cytb* and 53°C for *ND2*) for 45 s, and 72°C for 1 min, and a final 8 min at 72°C. The primer pairs used to amplify the six nuDNA loci were *Myo3F/Myo2*, *TGFB2.5F/TGFB2.6R*[70], *R50/R51*[71], *BRM15F/BRM15R*[72], *F2550/R2718*[73], and *VLDLR9F/VLDLR9R*[72]. PCR conditions were as follows: an initial denaturation at 94°C for 2 min, 35 cycles of 94°C for 30 s, annealing temperature (51°C for *BRM15*, 61°C for *RAG1* and 56°C for *MB*, *TGFB2*, *CHDa* and *VLDLR9*) for 30 s and 72°C for 30 s, and a final extension at 72°C for 10 min. Sequencing was carried out using the BigDye Terminator method (Applied Biosystems) on an ABI 3730xl analyzer. Complete sequences were assembled using Seqman II (DNASTAR) and compared visually to the original chromatograms to avoid reading errors. Sequences were aligned using CLUSTALW implemented in MEGA 5 [74]. To avoid amplifying mtDNA homologues from the nuclear genome (“numts”), we checked whether the sequences had ambiguous sites or stop codons. We phased heterozygous nuclear sequences using the program PHASE [75]. Only individuals with resulting phase probabilities greater than 0.7 were used in subsequent analyses [76,77]. All sequences have been deposited in GenBank (accession numbers KJ790262-KJ790672).

### Genetic diversity and phylogenetic analyses

We concatenated sequences of the two mtDNA fragments to a single sequence of 1908 base pairs. Fu and Li’ *D* test and Tajima’s *D* test implemented in DnaSP 5.0 [78] were used to assess whether nucleotide polymorphisms deviated from expectations under neutral theory for each locus in each species. Additionally, the multilocus HKA test was performed to test the neutrality of all loci using the HKA program [79]. DnaSP was used to calculate polymorphic sites, number of haplotypes, haplotype diversities and nucleotide diversities.

We used TCS 1.21 [80] to produce a maximum-parsimony network for each locus. The Bayesian algorithm implemented in BEAST 1.7.0 [81] was used to reconstruct phylogenetic relationships among mtDNA haplotypes. We selected the best-fit model of nucleotide substitution using the program MrModeltest 2.3 [82]. According to the Akaike information criterion (AIC), the best fit model for mtDNA was the HKY model [83]. The uncorrelated lognormal relaxed molecular clock and Yule process tree prior were chosen. Analysis was run for 20 million steps and sampled every 2,000 generations. We assessed convergence of the Markov chain Monte Carlo (MCMC) chain in the program Tracer 1.5 [84]. The output trees file was summarized into a maximum clade credibility tree using TreeAnnotator of the BEAST package. FigTree 1.3.1 [85] was used to visualize the result.

We used MEGA to calculate uncorrected *p*-distance between the two species at each locus. We also calculated the ratio of mtDNA to nuDNA divergence between the two species and other *Aegithalos* species pairs. Three other *Aegithalos* species (*A. niveogularis*, *A. concinnus* and *A. caudatus*) were used. We used two mtDNA fragments (*cytb* and *ND2*) and three nuDNA loci [*FIB* (beta-fibrinogen, intron 7), *TGFB2* and *ODC* (ornithine decarboxylase, intron 6–7)]. These species and loci were selected due to the accessibility in GenBank.

### Coalescent simulations

In order to distinguish between ILS and hybridization, we applied the coalescent-based IM model implemented in the program IMa [22]. The IM model involves several simplifying assumptions, such as no recombination within loci, mutation following the model applied, no population structure within species, and no genetic contribution from unsampled species [22,86]. A recent study has demonstrated that parameter estimates of the IM model are generally quite robust to small to moderate violations of the IM model assumptions [87]. We detected the minimum number of recombination events of each nuDNA locus using the four-gamete test in DnaSP. When recombination was detected, we kept the longest non-recombining block of sequences for subsequent analyses.

We conducted six IMa analyses. All analyses were first run in ‘M mode’. The HKY mutation model was used. The inheritance scalars were set as 1 for the three autosomal loci, 0.75 for the three Z-linked loci and 0.25 for the mtDNA to account for differences in effective population sizes. To improve mixing, we used a geometric heating scheme with six chains. We first performed multiple runs, with an increasing number of steps and using wide priors to ensure that the complete posterior distribution could be obtained. We finally performed three independent runs of 30 million steps with a burn-in period of three million steps. Convergence was assessed by monitoring trend plots, requiring all effective sample size (ESS) to be greater than 50, and ensuring the similarity of posterior distributions from independent runs. After M mode runs, we further ran IMa in ‘L mode’ to compare models with free or constrained migration.

### Demographic reconstruction

We conducted mismatch distribution analysis using Arlequin 3.5 [88]. Only the mtDNA alignment was used in this analysis. Individuals representing foreign mtDNA haplotype were excluded from this analysis. We also used the BSP method implemented in BEAST to estimate the posterior distributions of effective population size through time. Chains were run for 40 million generations, with the first 10% discarded as burn-in. The HKY model was used according to the result of MrModeltest. We used the uncorrelated lognormal relaxed molecular clock to account for rate variation among lineages. Demographic history through time was reconstructed with Tracer.

### Ecological niche modelling

We used MAXENT [89] to predict the potential distributions of both species at present and at the LGM. We downloaded 19 bioclimatic variables at 2.5 arc-minute resolution from WorldClim ( [90]; http://www.worldclim.org/). Species occurrence data included sampling sites, museum records (provided by the National Zoological Museum of China) and birdwatching locations (downloaded from http://birdtalker.net/report/). We removed sites separated from each other by less than 0.1° to reduce the effect of spatial autocorrelation. A total of 20 and 28 localities were used for *A. bonvaloti* and *A. fuliginosus*, respectively. Eighty per cent of the occurrence data were randomly selected to train the models and the remaining 20% were used to test the models. We set the number of maximum iterations to 2000 and the number of replicates to 10. The values of AUC and binomial probabilities were used to assess the models’ performance. To aid model interpretation, we used a conservative threshold—the lowest presence threshold [91] to distinguish ‘suitable’ from ‘unsuitable’ areas. The output maps were imported to ARC GIS 9.3 (ESRI, Redlands, CA) to produce final maps.

### Contemporary gene exchange

We used a Bayesian clustering method, implemented in NEWHYBRIDS [37] to assign to each individual the posterior probabilities of being pure *A. bonvaloti*, pure *A. fuliginosus*, F1 hybrid, F2 hybrid, backcross with *A. bonvaloti*, or backcross with *A. fuliginosus*. We ran the MCMC chain for 0.4 million iterations, with the first 10% as burn-in.

## Competing interests

The authors declare that they have no competing interests.

## Authors’ contributions

FL, CD and WW conceived and designed the experiments; CD, XY and GS collected samples; WW, CD and NZ performed the experiments; WW, CD and CZ analyzed the data; WW, PA and FL wrote the paper; YQ and SL provided valuable suggestions. All authors read and approved the final manuscript.

## Supplementary Material

Additional file 1 **The ratio of mtDNA to nuDNA divergence among five *Aegithalos *** **species pairs.** Click here for file

Additional file 2 **The marginal posterior probability density distributions of effective population sizes (Θ) and divergence times (*t*).** Click here for file

Additional file 3 Summary of likelihood ratio test statistics for the nested models analysis. Click here for file
