# Supplementary material for: Past hybridization between two East Asian long-tailed tits (Aegithalos bonvaloti and A. fuliginosus)
Source: Front Zool. 2014 May 16;11:40. doi: 10.1186/1742-9994-11-40 (PMC4040119; doi:10.1186/1742-9994-11-40)
Supplement: Additional file 1 — The ratio of mtDNA to nuDNA divergence among five Aegithalos species pairs. [file 1742-9994-11-40-S1.doc]

**Additional file**

Additional file 1 The ratio of mtDNA to nuDNA divergence among five *Aegithalos* speciespairs. Most sequences were downloaded from GenBank. The mtDNA and nuDNA divergence were calculated by averaging the divergences at each fragments/loci.

|  | *cytb* | *ND2* | *FIB* | *TGFB2* | *ODC* | mtDNA | nuDNA | mtDNA/nuDNA |
| --- | --- | --- | --- | --- | --- | --- | --- | --- |
| *bonvaloti - fuliginosus* | 0.002 | 0.001 | 0.006 | 0.004 | 0.004 | 0.002 | 0.005 | 0.4 |
| *bonvaloti - niveogularis* | 0.027 | — | 0.010 | 0.001 | 0.003 | 0.027 | 0.005 | 5.8 |
| *bonvaloti - concinnus* | 0.091 | 0.128 | 0.016 | 0.011 | 0.011 | 0.110 | 0.013 | 8.6 |
| *bonvaloti - caudatus* | 0.083 | 0.093 | 0.012 | 0.009 | 0.008 | 0.088 | 0.010 | 9.1 |
| *fuliginosus - niveogularis* | 0.029 | — | 0.007 | 0.006 | 0.007 | 0.029 | 0.007 | 4.4 |
| *fuliginosus - concinnus* | 0.088 | 0.130 | 0.012 | 0.011 | 0.011 | 0.109 | 0.011 | 9.6 |
| *fuliginosus - caudatus* | 0.084 | 0.094 | 0.008 | 0.009 | 0.008 | 0.089 | 0.008 | 10.7 |
| *niveogularis - concinnus* | 0.085 | — | 0.017 | 0.012 | 0.014 | 0.085 | 0.014 | 5.9 |
| *niveogularis - caudatus* | 0.078 | — | 0.014 | 0.010 | 0.012 | 0.078 | 0.012 | 6.5 |
| *concinnus - caudatus* | 0.106 | 0.137 | 0.018 | 0.016 | 0.015 | 0.122 | 0.016 | 7.4 |
