# Supplementary material for: Past hybridization between two East Asian long-tailed tits (Aegithalos bonvaloti and A. fuliginosus)
Source: Front Zool. 2014 May 16;11:40. doi: 10.1186/1742-9994-11-40 (PMC4040119; doi:10.1186/1742-9994-11-40)
Supplement: Additional file 2 — The marginal posterior probability density distributions of effective population sizes (Θ) and divergence times ( t ). [file 1742-9994-11-40-S2.doc]

Additional file 2 The marginal posterior probability density distributions of effective population sizes (*Θ*) and divergence times (*t*). All parameter estimates are scaled to the mutation rate. “all” and “allopatric” mean IM analyses based on all individuals and allopatric individuals, respectively.
