# Supplementary material for: Past hybridization between two East Asian long-tailed tits (Aegithalos bonvaloti and A. fuliginosus)
Source: Front Zool. 2014 May 16;11:40. doi: 10.1186/1742-9994-11-40 (PMC4040119; doi:10.1186/1742-9994-11-40)
Supplement: Additional file 3 — Summary of likelihood ratio test statistics for the nested models analysis. [file 1742-9994-11-40-S3.doc]

Additional file 3 Summary of likelihood ratio test statistics for the nested models analysis. 2LLR, 2*log likelihood ratio; df, degrees of freedom; *P*-value, significance level; *, *P* < 0.05; **, *P* < 0.01; ***, *P* < 0.001.

| Model | 2LLR | df | *P*-value |  | Model | 2LLR | df | *P*-value |
| --- | --- | --- | --- | --- | --- | --- | --- | --- |
| Mitochondrial DNA dataset from all individuals | | | |  | Mitochondrial DNA dataset from allopatric individuals | | | |
| ABCDD (mfb=mbf>0) | 1.829 | 1 | 0.176 |  | ABCDD (mfb=mbf>0) | -0.0047 | 1 | *P* >> 0.05 |
| ABCD0 (mfb>0, mbf=0) | −2.185 | 1 | *P* >> 0.05 |  | ABCD0 (mfb>0, mbf=0) | -0.0017 | 1 | *P* >> 0.05 |
| ABC0D (mfb=0, mbf>0) | 3.348 | 1 | 0.067 |  | ABC0D (mfb=0, mbf>0) | -0.0021 | 1 | *P* >> 0.05 |
| ABC00 (mfb=mbf=0) | 3.347 | 2 | 0.188 |  | ABC00 (mfb=mbf=0) | -0.0051 | 2 | *P* >> 0.05 |
| Autosomal DNA dataset from all individuals | | | |  | Autosomal DNA dataset from allopatric individuals | | | |
| ABCDD (mfb=mbf>0) | 1.891 | 1 | 0.169 |  | ABCDD (mfb=mbf>0) | 0.314 | 1 | 0.575 |
| ABCD0 (mfb>0, mbf=0) | 2.714 | 1 | 0.099 |  | ABCD0 (mfb>0, mbf=0) | 0.012 | 1 | 0.914 |
| ABC0D (mfb=0, mbf>0) | 4.652 | 1 | * |  | ABC0D (mfb=0, mbf>0) | 1.247 | 1 | 0.264 |
| ABC00 (mfb=mbf=0) | 10.094 | 2 | ** |  | ABC00 (mfb=mbf=0) | 6.015 | 2 | * |
| Z-linked DNA dataset from all individuals | | | |  | Z-linked DNA dataset from allopatric individuals | | | |
| ABCDD (mfb=mbf>0) | 4.098 | 1 | * |  | ABCDD (mfb=mbf>0) | 4.630 | 1 | * |
| ABCD0 (mfb>0, mbf=0) | 3.641 | 1 | 0.056 |  | ABCD0 (mfb>0, mbf=0) | 5.016 | 1 | * |
| ABC0D (mfb=0, mbf>0) | 0.166 | 1 | 0.683 |  | ABC0D (mfb=0, mbf>0) | 1.569 | 1 | 0.210 |
| ABC00 (mfb=mbf=0) | 8.664 | 2 | * |  | ABC00 (mfb=mbf=0) | 10.062 | 2 | ** |
